# Exploring integrative medicine for back and neck pain - a pragmatic randomised clinical pilot trial

**DOI:** 10.1186/1472-6882-9-33

**Published:** 2009-09-07

**Authors:** Tobias Sundberg, Max Petzold, Per Wändell, Anna Rydén, Torkel Falkenberg

**Affiliations:** 1 Unit for Studies of Integrative Health Care - Karolinska Institutet, Department of Neurobiology, Care Sciences and Society, Division of Nursing, 141 83 Huddinge, Sweden; 2 Nordic School of Public Health, PO Box 12133, 402 42 Göteborg, Sweden; 3 Center for Family and Community Medicine - Karolinska Institutet, Department of Neurobiology, Care Sciences and Society, 141 83 Huddinge, Sweden; 4 Health Care Research Unit - Sahlgrenska University Hospital, Bruna Stråket 30, 413 45 Göteborg, Sweden; 5 Vidar Clinic Foundation, Research Unit, 153 91 Järna, Sweden; 6 Scandinavian College of Chiropractic, Research Unit, 169 57 Solna, Sweden

## Abstract

**Background:**

A model for integrative medicine (IM) adapted to Swedish primary care was previously developed. The aim of this study was to explore the feasibility of a pragmatic randomised clinical trial to investigate the effectiveness of the IM model versus conventional primary care in the management of patients with non-specific back/neck pain. Specific objectives included the exploration of recruitment and retention rates, patient and care characteristics, clinical differences and effect sizes between groups, selected outcome measures and power calculations to inform the basis of a full-scale trial.

**Methods:**

Eighty patients with back/neck pain of at least two weeks duration were randomised to the two types of care. Outcome measures were standardised health related quality of life (the eight domains of SF-36) complemented by a set of exploratory "IM tailored" outcomes targeting self-rated disability, stress and well-being (0-10 scales); days in pain (0-14); and the use of analgesics and health care over the last two weeks (yes/no). Data on clinical management were derived from medical records. Outcome changes from baseline to follow-up after 16 weeks were used to explore the differences between the groups.

**Results:**

Seventy-five percent (80/107) of screened patients in general practice were eligible and feasible to enrol into the trial. Eighty-two percent (36/44) of the integrative and 75% (27/36) of the conventional care group completed follow-up after 16 weeks. Most patients had back/neck pain of at least three months duration. Conventional care typically comprised advice and prescription of analgesics, occasionally complemented with sick leave or a written referral to physiotherapy. IM care generally integrated seven treatment sessions from two different types of complementary therapies with conventional care over ten weeks. The study was underpowered to detect any statistically significant differences between the groups. One SF-36 domain showed a clinically relevant difference between groups that was also supported by a small distribution based effect size, i.e. vitality (-7.3 points, Cohen's *d *-0.34) which was in favour of IM. There was a clinical trend between groups showing that IM contributed to less use of prescription and non-prescription analgesics (-11.7 and - 9.7 percent units respectively) compared to conventional care. Exploring clinically relevant differences and the SF-36 as the basis for a main outcome measure showed that the sample sizes needed per arm to adequately power a full-scale trial depended on the target domain, i.e. ranging from 60 (vitality) to 339 (role emotion).

**Conclusion:**

This pilot study investigated the implementation of IM in the primary care management of non-specific back and neck pain. Recruiting patients and implementing IM in routine clinical practice was feasible. The results warrant further exploration into different perspectives and relevant combinations of outcome measures including the use of health resources, drugs and cost-effectiveness to help understand the relevance of IM in primary care. Future research should prioritise larger scale studies considering variability, pain duration and small to moderate treatment effects.

**Trial registration:**

Clinical trials NCT00565942

## Background

Research over the last few decades has reported an increased use of complementary therapies (CTs) [1-3] and an integration of CTs into mainstream medical settings, health care organisations and insurance plans [4-11]. These trends may present both new challenges and new opportunities for health care provision. In Sweden and elsewhere, major challenges include the great variety and quality of CT provision within health care and a lack of national and international recommendations of how integration of CTs with conventional care may be modelled [12]. This lack of conceptual models for delivering integrative medicine (IM) may partly be a result of a scarce evidence base in support of IM provision within public health care services. Most research studies have investigated different CTs in isolation, i.e. assessed specific efficacy of individual components in models of care. It remains largely unknown whether comprehensive IM models differ clinically or cost effectively from conventional care provision. Pragmatic trials investigating the comparative effectiveness of different models of care (each with several components) have been a reported priority to gain more understanding of IM in clinical practice [13,14].

Back and neck pain are conventionally managed in primary care, impose high costs, disability and decreased quality of life, and are two of the most common conditions treated by CTs [15-18]. Previously it has been found that IM in acute low back pain management, where a choice of massage, chiropractic or acupuncture was added to usual care, was as effective as usual care alone [19]. It is currently unknown if sub-acute to chronic back pain, for which conventional medical care is often costly and of limited benefit, respond differently to IM.

The current study was part of a research project exploring IM vs. conventional primary care on a health systems level, i.e. targeting general model effectiveness in a clinical context [20].

We have previously reported the results from the development of a comprehensive IM model adapted to Swedish primary care [21]. Consequently, the next phase of this project, and the aim of this pilot study was to explore the feasibility of conducting a randomised clinical trial to compare the effectiveness of the developed IM model with conventional primary care management, i.e. treatment as usual, for patients with non-specific back/neck pain of subacute to chronic duration. The specific objectives included analytical exploration of recruitment and retention rates, patient and care characteristics, clinical differences and effect sizes between groups, selected outcome measures and power calculations supported by collected data to inform the basis of a full-scale trial. Additional objectives were to test the feasibility of assessment, consent and data collection procedures.

## Methods

### Study design and setting

This explorative pilot study was conducted as a pragmatic randomised clinical trial, purposely in the setting of four primary care units in south suburban Stockholm. The implementation area could to some extent be characterised by a socio-economic status of higher unemployment rates, lower incomes and more welfare support and sickness benefits compared to the average levels in Stockholm [22].

### Patients and randomisation

Patients consulting general practitioners for back and/or neck pain were identified and screened for study inclusion by physicians in routine practice at participating primary care units from September 2004 to December 2005. Inclusion criteria were 18-65 years of age, low back and/or neck pain with or without headache of at least two weeks duration, resident of Stockholm County, literate in Swedish, willing to be randomised, and if allocated to IM attend up to 10 CT treatments during the study period and pay five Euros per treatment for the first six treatments after which no additional payment for CTs would be asked, i.e. a ceiling set at 30 Euros for patients to obtain all CT treatments in the study. Exclusion criteria included specific pathology and severe causes of back/neck pain such as malignant disease, vertebral fractures and severe or progressive neurological symptoms.

Potential participants were referred to the study's head general practitioner and telephoned by the research co-ordinator for a verification of eligibility, a study presentation and verbal informed consent of willingness to participate. Patients who fulfilled the inclusion criteria and had no exclusions were sent written study information, baseline questionnaires and an informed consent document. When the questionnaires and the written signed consent had been returned an assistant not involved in patient care randomly allocated the patient by a computer generated procedure with no blocking or stratification giving each participant equal probability to receive either continued usual care or the IM model of care. The research coordinator was informed about the allocation and then enrolled the patient.

### Interventions and providers

#### Conventional management

All patients first met their general practitioners to receive conventional care treatment plans. The conventional care complied with the clinical practice routines of the participating primary care units and the Stockholm county council management guidelines [23]. The guidelines for non-specific spinal pain disorders typically recommended advice (stay active), drug prescription (analgesics), sick leave (limited) and physiotherapy (activity based) [23]. There was no explicit study constraint to the provided conventional care as the study aimed to pragmatically reflect the general practitioners' standard care and treatment as usual. The general practitioners (n 35) of four neighbouring primary care units were invited to the study based on their involvement in back and neck pain management and interest to participate.

#### IM management

The model for delivering IM has been extensively described elsewhere [21]. In short, after the conventional care treatment plans had been advised, the IM management included consensus based team conferences to select and integrate relevant CTs into the management over a period of up to 12 weeks. The IM care was provided by a multidisciplinary IM team coordinated by a gate keeping general practitioner with clinical knowledge and experience of CTs and eight senior licensed/certified CT providers representing Swedish massage therapy, manipulative therapy*, shiatsu, acupuncture** and qigong, i.e. CTs with an emerging evidence base in general [24-33]. As treatments commenced, regular consensus case conferences followed, combining conventional and CT clinical reasoning in order to verify and improve the ongoing clinical management of the patient [21]. Patients did not participate during the consensus case conferences as it was considered more efficient for them to take part in the health care process by way of intermittent personal interaction with the IM provider team [21].

*Manipulative therapy was provided by a naprapath, one of several health care professions in Sweden utilising manipulation/mobilisation techniques to normalise joint and soft tissue dysfunction. Other common manipulative therapy providers in Sweden include osteopaths, chiropractors and orthopaedic manipulative physiotherapists. **A practitioner of traditional Chinese medicine provided acupuncture.

### Outcome measures

One of the research strategies prioritised by the IM team for this pilot study was to explore the feasibility of using an internationally standardised outcome measure in combination with a set of "IM tailored" outcomes in addition to qualitative exploration (data reported separately elsewhere) to adequately investigate the comprehensive IM intervention [21]. Here, the reliable and valid SF-36 questionnaire [34-36] was used to explore health-related quality of life domains (physical functioning, role physical, bodily pain, general health, vitality, social functioning, role emotional and mental health) where a score between 0 (worst) to 100 (best) was calculated for each dimension using standardised scoring systems. The IM tailored outcomes were eventually set to target self reported disability, stress, well-being, days in pain and the use of analgesics and health care. Numerical rating scales (0-10), where 0 indicated no and 10 indicated maximum levels respectively, were used to assess current disability of activities in daily living due to back/neck pain, stress and well-being. The disability rating scale was slightly modified from the second dimension of the reliable, valid and responsive Bournemouth questionnaire for back/neck pain [37,38]. The main modifications were to not specify the exact daily activities and asking for current instead of average disability, i.e. "How much does your back/neck pain interfere with your daily activities?" The stress and well-being scales were considered face valid and chosen to reflect common question areas targeted by CT providers in clinical practice. The number of days in pain during a fixed period of time has been valued an important indicator of pain persistence [39]. Based on group consensus and pre-testing of the selected outcome measures we stipulated an appropriate recall period of two weeks. The same time frame was chosen for assessing the face valid outcomes of self reported use (yes/no) of analgesics and health care in terms of prescription and non-prescription analgesics, conventional care and complementary care (CTs out of study). The outcome changes between baseline and follow-up after 16 weeks were used to explore and compare the results between the IM and conventional care groups.

### Data collection

Data were collected by postal questionnaires sent out two and six weeks post the intervention period, i.e. after 12 and 16 weeks respectively. The administration of questionnaires, including up to two reminders, were managed outside of the primary care units by research staff not involved in the clinical care of patients. Hence all providers of care were blinded to the patient questionnaires but not to treatment allocation. The patients' general practitioners documented the conventional care treatment plans whereas the IM team documented data on IM care in a separate medical record specifically developed for the study.

### Statistical procedures

Quality of life data (SF-36 questionnaires) were digitally scanned by standardised procedures at Göteborgs Universitet (HRQL gruppen, Göteborg, Sweden). Other outcome data were double entered into EpiData 3.1 (EpiData Association, Odense, Denmark) by research assistants not involved in the clinical care of patients. After cleaning and validation the raw data was imported into Intercooled Stata™ 9.2, (StataCorp, College Station, TX, USA) and SPSS™ 15.0 (SPSS Inc, Chicago, IL, USA) for statistical analyses.

Numerical variables were summarised as means, standard deviations, mean differences and proportions of change between baseline and follow-up after 16 weeks. The Mann-Whitney test was used for statistical analyses of differences between the groups for ordinal data (numerical rating scales and quality of life) and the independent two sample t-test was used for analysing days in pain. Dichotomous variables were summarised as the proportions of patients using the different types of analgesics and health care at the different time points.

When testing cross-sectional group differences at baseline we used univariate analysis where the different outcome measurements were dependent variables and the group allocation independent. When assessing the differences in change over time between groups we used a multiple regression model with time point, group allocation and the interaction term (equal to 1 if the measurement was from the IM-group and taken at follow up, and equal to 0 otherwise). We chose to not adjust for other covariates in the analysis. For the analysis of difference in change over time between the groups a mixed model was applied to account for repeated values within patients. The difference was estimated as the interaction term being 1 if the measurement was from the IM-group and taken at follow-up, and 0 otherwise.

All patients were kept in their assigned groups. Patients lost to follow-up, i.e. observations with missing data, were excluded from the primary analyses. To comply with a more comprehensive intention-to-treat strategy we did a secondary analysis where the last observed measures were imputed for missing data. A significance level of 5% was used and 95% confidence intervals were reported. All p-value calculations were two-tailed.

No previous data about our specific study population were available and the sample size for the pilot trial was based on a hypothesis of disability scores (exploratory outcome). The hypothesis included that 0-10 ratings of disability would be about equal in the two groups at baseline, around 5. It was decided to power the study to detect a mean improvement of at least 2 points in the integrative medicine group vs. 1 point in the conventional care group. This difference was hypothesised to be of clinical relevance in order for the IM model to be advisable at follow-up after 16 weeks. Applying 80% power, significance level of 5% and assuming a standard deviation of 1.5 gave a sample size of 36 patients per treatment arm (n = 72) (STATA software). The sample size was increased to n = 80, which was reasoned sufficient to give an initial estimate of proportion for this type of explorative study.

The research project was approved by the regional ethics committee at Karolinska Institutet (Dnr: 668-03, 650-04 and 121-32).

## Results

### Recruitment and retention

**Figure 1:**
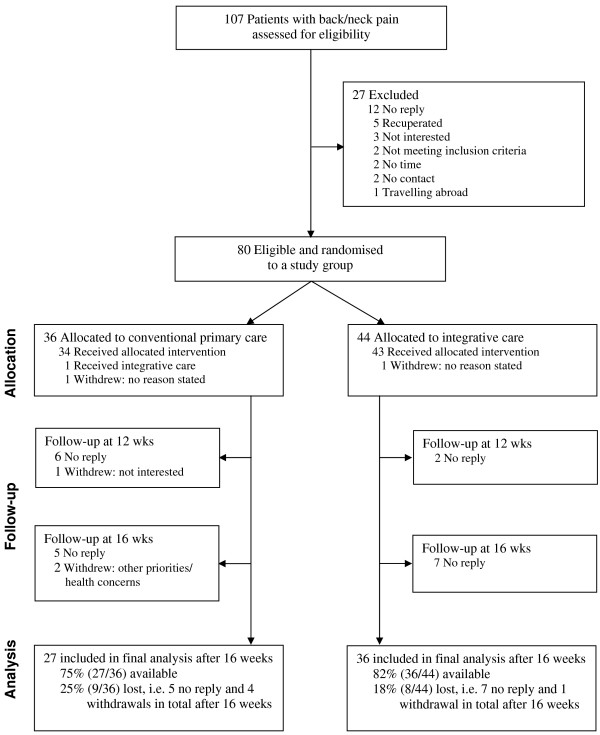
**CONSORT flow-chart**. The flow of patients through the randomised clinical trial (CONSORT flow-chart).

About 75% of patients seeking help for back/neck pain at the primary care units were eligible and feasible to include in the trial, i.e. in total 107 patients were assessed for eligibility to be able to include 80 patients in the study (Figure 1). Demographics and baseline characteristics were similar between the groups, most patients had back/neck pain of at least 3 months duration, and there were no statistically significant differences between the groups (Table 1). Of the recruited patients 82% (36/44) in the IM group and 75% (27/36) in the conventional care group completed follow-up after 16 weeks (Figure 1). There were nine or 25% (9/36) dropouts in the conventional care group and eight or 18% (8/44) dropouts in the IM group after 16 weeks (Figure 1). Age and gender characteristics of dropouts were similar between the groups, i.e. conventional care group (mean age 41 ± 9.7 years, seven women) and IM group (mean age 38 ± 9.2 years, five women) and there were no significant differences in outcome characteristics.

**Table 1:** Baseline characteristics of study participants by randomised groups

	**Conventional care (n = 36)**	**Integrative care (n = 44)**
Age, mean (SD)	41.1 (10.4)	40.3 (9.4)
Female, %	72%	73%
EU nationality, %	89%	81%
Location of worst pain, %		
Low back	53%	52%
Neck	33%	36%
Low back and neck	14%	11%
Duration of pain, %		
Two weeks to three months	17%	12%
Three months or longer	83%	88%
Days with pain over the last two weeks (0-14), mean (SD)	12.1 (2.8)	11.8 (3.8)
Disability due to back/neck pain (0-10)^*a*^, mean (SD)	5.4 (2.6)	5.4 (3.0)
Stress (0-10)^*a*^, mean (SD)	5.2 (2.7)	5.6 (2.7)
Wellbeing (0-10)^*a*^, mean (SD)	4.8 (1.9)	5.1 (2.3)
Used prescription analgesics during the last two weeks, % yes	54%	45%
Used non-prescription analgesics during the last two weeks, % yes	57%	63%
Used conventional care during the last two weeks, % yes	61%	65%
Used complementary care over the last two weeks, % yes	26%	20%
SF-36 Health related quality of life^*b*^		
Physical functioning, mean (SD)	69.4 (17.3)	70.1 (24.4)
Role physical, mean (SD)	21.5 (33.4)	29.0 (35.3)
Bodily pain, mean (SD)	32.0 (14.5)	34.0 (19.1)
General health, mean (SD)	55.1 (18.6)	56.4 (24.0)
Vitality, mean (SD)	36.4 (16.6)	32.3 (23.3)
Social functioning, mean (SD)	61.5 (24.2)	56.3 (30.0)
Role emotional, mean (SD)	54.2 (39.9)	58.3 (43.2)
Mental health, mean (SD)	63.1 (19.3)	61.1 (21.2)

^*a*^The anchors for the numerical ratings scales were 0 (nothing) to 10 (maximum) levels of disability, stress and wellbeing respectively. ^*b*^SF-36 quality of life domains, min-max score from 0 (worst) to 100 (best). SD, standard deviation. There were no statistically significant differences between the randomised groups.

About 80% of physicians from the same primary care unit as the head general practitioner referred patients to the trial compared to about 10% of general practitioners of the other primary care units.

### Outcome measures

#### Clinical differences and effect sizes between groups

One SF-36 domain showed a clinically relevant difference between groups [40,41] that was also supported by a small distribution based effect size [42], i.e. vitality (-7.3 points, Cohen's *d *-0.34) which was in favour of IM (Table 2). Self-rated disability and stress returned small clinical differences (0.7 and 1.2 points) and small effect sizes (Cohen's *d *0.23 and 0.43) between groups supporting IM (Table 2). The clinical use of drugs and health resources showed a trend between groups that IM contributed to less use of prescription and non-prescription analgesics (-11.7 and - 9.7 percent units respectively) compared conventional care (Table 3).

**Table 2:** Change in SF-36, numerical rating scales and days in pain from baseline to follow-up after 16 weeks

	**Conventional care**		**Integrative care**		**Conventional vs. integrative**		
	
**Outcome measure**	**Mean (SD)**	**N**	**Mean (SD)**	**N**	**Diff**.	***d***	**P**
Physical functioning*	11.0 (16.8)	27	9.1 (13.2)	35	1.9	0,09	0.920

Role physical*	28.7 (46.9)	27	29.2 (39.4)	36	-0.5	-0,01	0.875

Bodily pain*	19.1 (22.5)	27	21.2 (23.4)	36	-2.1	-0,12	0.813

General health*	7.5 (21.0)	26	6.1 (10.8)	36	1.4	0,06	0.502

Vitality*	12.1 (16.6)	27	19.4 (21.8)	35	-7.3	-0,34	0.237

Social functioning*	13.4 (21.9)	27	14.6 (21.0)	36	-1.2	-0,04	0.703

Role emotional*	16.1 (44.7)	27	8.3 (48.1)	36	7.7	0,18	0.872

Mental health*	5.6 (18.9)	27	7.3 (16.0)	35	-1.7	-0,09	0.326

Disability (a)	-1.2 (3.5)	26	-1.9 (2.9)	36	0.7	0,23	0.458

Stress (b)	0.2 (2.8)	26	-0.9 (2.2)	36	1.2	0,43	0.090

Well-being (c)	1.5 (2.1)	26	1.5 (2.1)	36	-0.1	-0,03	0.873

Days with pain (d)	-3.1 (4.7)	26	-3.8 (5.5)	35	0.7	0,19	0.595

*SF-36 health domains, min-max score from 0 (worst) to 100 (best). Numerical rating scales targeting; (a) disability in activities of daily living due to back/neck pain; (b) perceived stress; (c) well-being. The anchors for the numerical ratings scales were 0 (nothing) to 10 (maximum) levels of disability, stress and well-being respectively. (d) Days with pain over the last two weeks (0-14). SD, standard deviation. Diff, clinical difference between groups in outcome change over time (suggested magnitude for SF-36; 2 small, 5 clinically relevant, 10 moderate, 20 large [40,41]). d, effect size by Cohen's d (0.20 small, 0.50 moderate and 0.80 large [42]). Statistical analyses by Mann-Whitney (numerical rating scales and SF-36) and independent two sample t-tests (days with pain).

**Table 3:** Change in self reported use of analgesics and health care from baseline to follow-up after 16 weeks

	**Conventional care**			**Integrative care**			**Conventional vs. integrative care**	
	
**Type of health resource**	**From % (n)**	**To % (n)**	**Percent units**	**From % (n)**	**To % (n)**	**Percent units**	**Diff**.	**P OR (95% CI)**
Prescription analgesics	54.3% (19/35)	40.0% (10/25)	-14.3	45.5% (20/44)	19.4% (7/36)	-26.0	-11.7	0.325 0.34 (0.0 to 3.0)

Non-prescription analgesics	57.1% (20/35)	42.3% (11/26)	-14.8	63.4% (26/41)	38.9% (14/36)	-24.5	-9.7	0.703 0.67 (0.1 to 5.2)

Conventional care	61.1% (22/36)	15.4% (4/26)	-45.7	65.1% (28/43)	22.2% (8/36)	-42.9	2.8	0.720 1.38 (0.2 to 8.0)

Complementary care	25.7% (9/35)	33.3% (8/24)	7.6	19.5% (8/41)	19.4% (7/36)	-0.1	7.5	0.762 0.75 (0.1 to 4.8)

All measures were for the self reported use over the last two weeks. Diff, difference in change over time between groups. OR, Odd's ratio. CI, confidence interval.

#### Statistical differences, power analysis and sample size

Both the IM and conventional primary care groups improved over time (Tables 2 and 3). The primary analysis showed that there was generally large response variability and that the study was underpowered to detect statistically significant differences between the groups for any of the selected outcome measures (Tables 2 and 3). The secondary analysis (intention-to-treat) where the last observed measures were imputed for missing data did not change the results with lack of statistically significant differences between the groups (data not shown).

Exploring clinically relevant differences and the use of SF-36 as the basis for a main outcome measure in a full-scale trial, employing 80% power, a significance level of 5%, a 10 points clinical difference in change over time between groups [40,41] and the standard deviations derived from our pilot data, showed that the sample size needed per arm would range between 60 (vitality) to 339 (role emotional) for the trial to be adequately powered in relation to the main target domain.

### Characteristics of care

The conventional care treatment plans were characterised by advice (85%), prescription of analgesics (50%), sometimes complemented with short-term sick leave (33%) or a written referral to physiotherapy (26%). The advice was given by the general practitioner and could be categorised into general, e.g. to stay active (33%); the use of analgesics (15%); or recommendations about physiotherapy (37%). The patients in the IM group additionally attended seven CT treatment sessions administered over a time period of ten weeks (Table 4). The division between western/body based CTs and eastern/energy based CTs were similar (Table 4). Among the energy based CTs acupuncture was less used than shiatsu (Table 4). On average the patients attended one session of individualised activity based self-help in the form of qigong (Table 4). Typically the patients received two different types of CTs (Table 5). Swedish massage was the most common therapy to be combined with several other types of CTs. Shiatsu and qigong were the two most common CTs to be combined together (Table 5). Manipulative therapy was the most common CT to be integrated as a single add-on treatment to conventional care (Table 5). There were no reports of crucial adverse events with either type of care.

**Table 4:** Categories, types and numbers of complementary treatments provided in the IM model during the treatment period

**Western, body based**		**Eastern, energy based**		**Self help, activity based**
**Swedish massage**	Manipulative therapy*	**Shiatsu**	Acupuncture**	**Qigong**

1.5 (2.7)	1.8 (2.6)	2.8 (3.4)	0.3 (1.6)	1.0 (1.8)
	
1.6 (2.6)		1.5 (2.9)		

7.3 (3.2)				

Table figures refer to mean (standard deviation) estimates. The average length of the treatment period was 10.2 (1.4) weeks. *Manipulative therapy was provided by a naprapath. **A practitioner of traditional Chinese medicine provided acupuncture.

**Table 5:** Combinations of complementary therapies provided in the IM model

**Categories**	**Types**	**Different CTs (n)**	**Patients % (n)**
Western	Swedish massage	1	6.8 (3)
	
	Manipulative therapy*	1	22.7 (10)

Eastern	Shiatsu	1	15.9 (7)
	
	Acupuncture**	1	4.5 (2)

Western + Eastern	Manipulative therapy* + Shiatsu	2	11.4 (5)
	
	Swedish massage + Shiatsu	2	9.1 (4)
	
	Swedish massage + Acupuncture**	2	2.3 (1)

Eastern + Self help	Shiatsu + Qigong	2	13.6 (6)

Western + Self help	Swedish massage + Qigong	2	6.8 (3)
	
	Manipulative therapy* + Qigong	2	2.3 (1)

Western + Western	Swedish massage + Manipulative therapy*	2	2.3 (1)

Western + Eastern + Self help	Manipulative therapy* + Shiatsu + Qigong	3	2.3 (1)
	
		Total	100.0% (44)

Western, body based therapies: Swedish massage and manipulative therapy*. Eastern, energy based therapies: Shiatsu and acupuncture**. Self help, activity based therapy: Qigong. *Manipulative therapy was provided by a naprapath. **A practitioner of traditional Chinese medicine provided acupuncture.

The crude costs for the CT treatments in the IM model were approximated to 365 € (3320 SEK). These direct costs were calculated from estimates of non-reimbursed CT treatment costs in Sweden (figures derived from CT associations/representatives), i.e. Swedish massage 44 € (400 SEK), manipulative therapy 57 € (520 SEK), shiatsu 53 € (480 SEK), acupuncture 35 € (320 SEK), and qigong 48 € (440 SEK) in relation to the average number of CT treatments provided in the IM model (Table 4).

### Assessment, consent and data collection procedures

Assessment and data collection procedures were feasible throughout the study. This included the physicians' screening of potential study participants during routine clinical practice, the referral to the head general practitioner and the verification of eligibility and obtaining oral and written informed consent. However, it was not possible to verify the total number of patients screened at the participating primary care units. Similarly, logistical barriers such as incompatibility between different providers' documentation and electronic patient record systems hindered mutual access to records of conventional or complementary care provided outside of the IM-team. Documentation of IM care was also challenging, e.g. in terms of standardisation of recording procedures, patient record contents and mutual terminology.

## Discussion

### Recruitment and retention

The patient recruitment process was feasible and resulted in a high success rate, i.e. 75% (80/107) of screened patients with back/neck pain were enrolled in the trial. Facilitators may have been general practitioner assurance, e.g. enforcing that they only had to screen regular patients and did not have to engage in the practicalities of enrolling the patients. The recruitment rate may however be an over estimation due to logistical barriers which prevented us from making direct comparison of the reported number of screened patients with the actual number of patients with back/neck pain having sought care at the participating primary care units. The head general practitioner's personal experience of professional CT provision, e.g. Swedish massage and acupuncture, may have been an additional recruitment facilitator. This was known to several of the collaborating physicians and may have increased their trust in the project and willingness to refer patients. Adding to this we found that physicians who worked at the same primary care unit as the head general practitioner referred the most patients to the trial. Perhaps regular informal contact among colleagues facilitated opportunities for queries and constructive dialogues in familiar biomedical terms about the rational for IM in primary care. Communicating in the same "language" in relation to biomedicine and CTs has indeed been suggested to increase understanding across different health care disciplines [43,44].

Patients lost to follow-up displayed similar characteristics between groups and were mostly due to no reply and attained 25% (9/36) for conventional care and 18% (8/44) for IM care after 16 weeks. One retention barrier may have been the use of postal questionnaires administered outside of the primary care units to collect data. Follow-up strategies "closer" to the patients, e.g. on-site questionnaires or interviews by dedicated staff members not involved in the clinical care of patients or follow-up procedures via telephone, might have been more feasible. However, a prerequisite for such strategies include the availability of resources and adequate funding which the current study did not have [21]. Additional facilitators for achieving high retention rates may include recruiting patients via media advertising or different health related registers, businesses or insurance companies. However, we decided against such external recruitment approaches reasoning that patients enrolled through active media advertising may behave differently (e.g. more motivated), have different socioeconomic characteristics and intentions for participating (e.g. more positive towards CTs) compared to the patients seeking regular care at their primary care units in a socioeconomically underpoverished suburban area. Hence the selected recruitment strategy may have increased the generalisability of findings in relation to the target population and clinical setting in the particular area, i.e. high external validity.

No patients dropped out due to crucial adverse effects with either type of care or due to the slight extra cost of receiving CTs in the IM model. This supports the IM and conventional care models as safe treatment options, as well as the feasibility of letting patients partly contribute to the costs for CT provision. This may have important clinical implications for implementation and sustainability of IM in public health care settings where CTs are not normally reimbursed. Future studies will have to investigate at what levels the economical thresholds lies and how much patients are willing to financially contribute for IM health services in different clinical settings.

### Characteristics and costs of care

Conventional care provision complied with the recommended primary care guidelines [23]. To this the IM model integrated seven CT sessions over ten weeks, typically from two different CT types. The average number of CT treatments was about equally distributed between the western/body based and eastern/energy based CT categories. Acupuncture was the least provided CT, mainly due to difficulties recruiting an acupuncturist. The IM team compensated for this by providing shiatsu, a CT largely sharing the philosophy of acupuncture. The focus on self-help, activity based qigong therapy was an important component of the IM model that may have additional social/behavioural aspects worth exploring in future trials. Manipulative therapy was provided by a naprapath, a common provider of this type of care in Sweden. Future IM approaches may want to consider other recognised manipulative therapy professions including e.g. osteopaths, chiropractors and orthopaedic manipulative physiotherapists to facilitate the recruitment of manipulative therapy providers to IM teams.

The extra direct costs for CT treatments for achieving these clinical results was estimated to 365 € (3320 SEK) in total. However, this limited cost approximation did not include data on e.g. costs for conventional care, administration, case conferences, indirect costs or cost savings from patient, provider or organisational perspectives. Future studies are warranted and should consider collection of more detailed cost related data, e.g. by cost diaries and data base measures of health care visits and sick leave, to gain a thorough understanding about the costs and cost-effectiveness of IM vs. conventional care. Such evidence has immediate implications for health policy and decision-making regarding the implementation of IM in conventional primary care.

### Outcomes

The pilot trial analyses did not result in any statistically significant differences between the groups' outcome changes over time. Imputing missing data with the last observed measures (intention-to-treat) did not change the results, i.e. indicating that the chosen strategy was robust for excluding cases with missing data. Exploring outcome differences and effect sizes between the groups returned few clinically relevant differences. The outcome measures that displayed the largest clinical differences supporting IM, albeit within small ranges, were the SF-36 quality of life domain vitality and the decreased use of prescription and non-prescription analgesics. Clearly, less use of prescription analgesics if confirmed is an important clinical finding that may reduce reported negative side effects linked to prolonged use of such drugs [45,46]. Other small trends in favour of IM were seen in two of the exploratory IM tailored outcomes in terms of decreased disability and less stress. Perhaps the IM model due to the individualisation of back/neck pain management by integration of CTs facilitated additional and more personalized ways of supporting and empowering patients compared to possibilities within conventional care. However, the added CT treatments for the IM group might have exposed those patients to a more intensive management, which in turn may help to explain the trend towards more positive results for IM identified for some variables. This increased "attention" effect is purposively part of the IM model and allowed for in pragmatic and exploratory approaches towards investigating differences between models of care. The concurrent use of CTs out of the study allowed for in this pragmatic pilot study, although not actively recommended to patients (we simply monitored their self-reported use), may have contaminated the trial and contributed to the lack of significant differences between groups. Nonetheless, to inflict or restrict self-initiated health care strategies or utilisation patterns, or to quantify and distinguish between placebo or non-specific effects of attention from more specific effects of e.g. isolated CT technologies or procedures, future studies would have to use more explanatory designs. Although such rather costly and complicated investigations would provide high internal validity, the generalisability of findings from such trials to regular primary health care provision can sometimes be questioned [20].

The current results with generally small clinical differences and effect sizes between groups may challenge a narrow use of outcomes measures in isolation to understand the relevance of IM in primary care. The findings may further attest to the need of identifying additional more relevant evaluation strategies, as suggested by recent outcomes research targeting IM and complex health interventions [47]. The trend of decreased use of prescription and non-prescription analgesics for IM in this pilot trial may support that the use of drugs and health care resources might indeed be one such important target area. Aspects of prevention, lifestyle changes and health promotion are other potentially important areas suggested, e.g. in recent research on shiatsu [48], incidentally one of the CTs in our IM model. Lastly, an iterative cycle integrating and triangulating complementing quantitative and qualitative investigative procedures might be one of the best approaches towards exploring complex interventions such as the implementation of IM in clinical care settings [49].

### Summary of strengths and limitations

Strong points in this study were successful screening and recruitment procedures; feasible CT provision within conventional care; comprehensive results on the characteristics of IM care with expected high external validity; acquisition of data to explore statistical and clinical differences between groups and to adequately power a future large-scale trial. Limitations included a relatively high drop out rate after 16 weeks; underestimated variability and lack of power to detect statistically significant differences between the groups; no blocking in the randomisation procedure; several outcome measures of explorative nature lacking proper scientific validity; scarce results on the use of health care resources, costs and cost savings. We have discussed some implications of this for future research strategies including the need to continue investigations into relevant combinations of outcomes measures to adequately target and understand the relevance of IM for back and neck pain management in primary care settings.

## Conclusion

It has been proposed that for many patients the process of care may be as important as the outcomes of treatment [50] which may explain in part the relatively large utilisation of CTs among consumers and patients globally [51]. Indeed, care processes themselves may influence care outcomes, not merely in terms of satisfaction with care but also with respect to patients' state of health and treatment effectiveness [50]. Accordingly, the IM model emphasized care processes along with care outcomes, and considering some of the clinical trends reported here, aspects of IM care might be important to consider in primary health care reform. In conclusion this pilot study has demonstrated the feasibility of conducting a randomised clinical trial comparing a model for IM with conventional primary care management of patients with non-specific back/neck pain of mostly chronic duration. Recruiting regular primary care patients in routine clinical practice was feasible. Exploring clinically relevant differences and the use of SF-36 as the basis for a main outcome measure showed that the sample sizes needed per arm would range from 60 (vitality) to 339 (role emotion) to adequately power a full-scale trial. The findings attest to the need to further investigate IM as a complex health intervention and to continue to explore relevant combinations of outcomes to help understand the relevance of IM in primary care, e.g. by including patient and provider perspectives, detailed use of drugs and health care resources and health economic evaluations. Future research should prioritise larger scale studies considering variability, pain duration and small to moderate treatment effects.

## Competing interests

Tobias Sundberg and Torkel Falkenberg are affiliated with the Unit for Studies of Integrative Health Care at Karolinska Institutet. Tobias Sundberg was financed by a grant from the Foundation for Manual Therapy Research (Insamlingsstiftelsen för forskning om manuella terapier) during the IM project. Per Wändell, Max Petzold and Anna Rydén declare no competing interests. The IM project was partly funded by the following Swedish organisations: Ekhagastiftelsen, Insamlingsstiftelsen för forskning om manuella terapier, and Svensk förening för vetenskaplig homeopati. The funding agencies did not have any influence on the design and conduct of the study; collection, management, analysis, and interpretation of the data; and preparation, review, or approval of the manuscript.

## Authors' contributions

TS drafted the original manuscript and participated in redrafting and rewriting. TF conceived the general research design, critically read the manuscript and participated in redrafting and rewriting. MP outlined the statistical analyses, critically read the manuscript and participated in rewriting. PW provided primary care aspects, critically read the manuscript and participated in rewriting. AR helped with quality of life analyses, critically read the manuscript and participated in rewriting. All authors read and approved the final manuscript.

## Pre-publication history

The pre-publication history for this paper can be accessed here:


